# Serum level of DKK-1 and its prognostic potential in non–small cell lung cancer

**DOI:** 10.1186/1746-1596-9-52

**Published:** 2014-03-10

**Authors:** Liang-liang Dong, Lu-yun Qu, Li-yan Chu, Xiao-hui Zhang, Ying-hui Liu

**Affiliations:** 1 Department of Medical Oncology, Yantai Yuhuangding Hospital, 20 Yuhuangding East Road, Yantai 264000, China

**Keywords:** Dickkopf-1, Non-small cell lung cancer, Prognosis

## Abstract

**Background:**

The aim of the present study was to measure the serum level of dickkopf-1(DKK-1) in patients with non-small cell lung cancer (NSCLC), and to determine the prognostic potential of serum DKK-1 in NSCLC.

**Material and methods:**

The present study included a total of 150 patients with NSCLC and 150 healthy controls. Serum level of DKK-1 was measured by enzyme-linked immunosorbent assay (ELISA). Numerical variables were recorded as means ± standard deviation (SD) and analyzed by independent t-tests. Categorical variables were presented as rates and analyzed by using the chi-square test or Fisher’s exact test. The overall survival was analyzed by log-rank test, and survival curves were plotted according to Kaplan–Meier.

**Results:**

We found that serum DKK-1 level was significantly higher in patients with NSCLC than healthy controls. Mean serum DKK-1 level was 31.42 ± 6.32 ng/ml in the NSCLC group and 14.12 ± 3.29 ng/ml in the healthy control group (p <0.01). Serum DKK-1 level expression level was significantly positively correlated with TNM stage (p = 0.009), lymph node involvement(p = 0.001), and distant metastases(p < 0.001).

In the multivariate Cox proportional hazards analysis, high DKK-1 expression was independently associated with poor survival (P < 0.001; HR = 3.98; 95% CI =2.19-4.83).

**Conclusions:**

In conclusion, our results showed that DKK-1 was overexpressed in NSCLC, and DKK-1 in serum was a good predictor of poor prognosis in patients with NSCLC. More researches are needed in the future to clarify the detailed mechanism of DKK-1 in the carcinogenesis and metastasis of NSCLC.

**Virtual slides:**

The virtual slides for this article can be found here: http://www.diagnosticpathology.diagnomx.eu/vs/1471414150119415.

## Introduction

Lung cancer is one of the leading causes of all cancer related deaths worldwide, with a 5-year survival as low as 13% [1]. Non small cell lung cancer (NSCLC) represents approximately 85% of lung cancer cases and comprises several histological phenotypes, the most common being adenocarcinoma, squamous-cell carcinoma and large-cell carcinoma. When dealing with NSCLC, we still face a lot of clinical problems. Most patients have locally advanced or metastatic disease at the time of diagnosis, and only a third of NSCLC cases are considered technically and oncologically treatable with a radical surgical intervention [2]. The overall prognosis of NSCLC is poor, because it exhibits high resistance to anticancer therapy [1]. A promising breakthrough to improve the outcome for NSCLC patients is the introduction of validated biomarkers into clinical management. These may be crucial not only for early diagnosis but also to assist treatment choice for the most optimal therapeutic interventions.

Previous studies have shown that the Wnt signaling pathway regulated proliferation, fate specification, polarity and migration of cells [3,4]. The Dickkopf (DKK) family of proteins are known as antagonists for the Wnt-β-catenin signalling pathway, which includes DKK-1, DKK-2, DKK-3 and DKK-4 [5]. DKK-1 encodes a secreted Wnt antagonist that binds to LRP5/6 and so induces its endocytosis, leading to the inhibition of the canonical pathway [6]. DKK-1 itself is a target of the beta- catenin/TCF signaling pathway [7]. Previous studies have shown that the expression of DKK-1 was down-regulated significantly in human colon cancer, gastric cancer and melanoma [7-9]. However, paradoxically, DKK-1 has been found to be overexpressed in hepatoblastomas, hepatocellular carcinomas, and Wilms’ tumors [10,11], suggesting that the function of DKK-1 may be different in different cancers. Previous studies have investigated the expression and functions of several proteins in lung cancer [12-16]. However, the expression level of DKK-1 in primary lung cancer and its relationship with clinicopathological factors has not been examined, therefore, and the biological roles of DKK-1 in lung cancer cells are still unclear.

In the present study, we measured the serum levels of DKK-1 in patients with NSCLC and healthy controls. We sought to determine the prognostic potential of DKK-1 in NSCLC.

## Materials and methods

### Patient, healthy controls, and serum samples

The selection criteria for patients with NSCLC were as follows: (1) pathologically confirmed patients with NSCLC(the diagnoses in all patients were confirmed each time by microscopic examination of the material obtained during bronchoscopy, biopsy, and/or surgery); (2) the patients had no history of other cancers. A total of 150 patients with NSCLC in Yantai Yuhuangding Hospital between June 2006 and July 2012 were enrolled in the present study. All subjects underwent clinical examination; plain chest radiograph; CT scan of the chest, upper abdomen, and brain; fiberoptic bronchoscopy; and bone scan. Blood samples were collected from the patients at the time of diagnosis, before any kind of treatment (surgery, radiation, or chemotherapy). The demographic and pathological data, including age, gender, and the tumor stage were obtained by a review of the patients’ medical records (the data was used with the consent of the patients as well as the approval of the Ethics Committee of Yantai Yuhuangding Hospital). Tumor stage was determined according to the 2009 TNM staging classification system. Fasting blood was taken for all participants and serum was collected and stored at −80°C.

### Enzyme-linked immunosorbent assay

Serum DKK-1 levels were measured by enzyme-linked immunosorbent assay (ELISA) with immunoassay kit (Miltenyi, Germany) according to the manufacturer’s directions. The optical density (OD) at 450 nm was determined. The standard curves were established with OD450 as Y axle and the concentration of standard substance as X axle. The level of protein was obtained through standard curve. Results were reported as concentration of DKK-1 ng/ml in samples.

### Statistical analysis

Statistical analyses were performed using SPSS 13.0 soft-ware (Chicago, Ill., U SA) and GraphPad Prism 5 (GraphPad Software Inc., CA, USA). Numerical variables were recorded as means ± SD and analyzed by independent t-tests. Categorical variables were presented as rates and analyzed by using the chi-square test or Fisher’s exact test. The overall survival was analyzed by log-rank test, and survival curves were plotted according to Kaplan–Meier. Univariate Cox regression was performed on each clinical covariate to examine its influence on patient survival. Final multivariate models were based on step-wise addition. P-values <0.05 was considered as statistically significant.

## Results

### Clinical features of patients with NSCLC and healthy controls

150 patients with recently diagnosed NSCLC were enrolled in the present study, and 150 healthy persons were used as a control group. Among the 150 patients with NSCLC, 65 patients were treated with surgery. Mean age was 64.2 ± 15.1 yr in the NSCLC group and 62.1 ± 14.3 yr in the control group (P>0.05). There were 81 males and 69 females in the NSCLC group, and 77 males and 73 females in the control group (P>0.05). The clinical features of 150 patients with NSCLC and 150 controls was summarized in Table 1.

**Table 1:** Clinical features of patients with non–small cell lung cancer and controls

**Variables**	**Patients (N = 150)**	**Controls (n = 150)**	**P value**
Age			
Mean (SD), yr	64.2 ± 15.1	62.1 ± 14.3	
< 50 yr	15	18	
50-70 yr	85	83	
> 70 yr	50	49	>0.05
Sex			
Male	81	77	
Female	69	73	>0.05

N: number; SD: Standard Deviation.

### The serum level of DKK-1 in patients with NSCLC and controls

**Figure 1:**
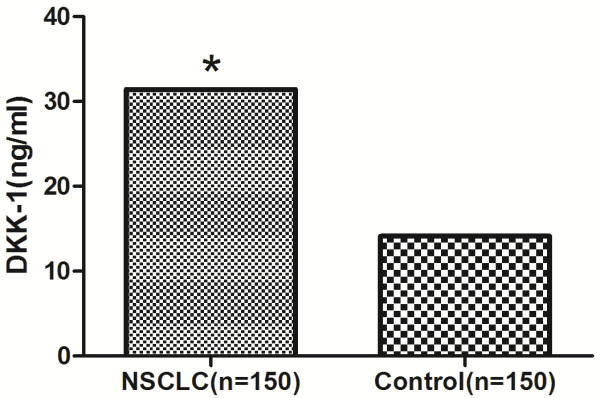
**Serum DKK-1 levels in patients with NSCLC and controls.** Mean serum DKK-1 level was 31.42 ± 6.32 ng/ml in the NSCLC group and 14.12 ± 3.29 ng/ml in the control group (p <0.01).

Serum DKK-1 level was found to be significantly higher in patients with NSCLC than controls. Mean serum DKK-1 level was 31.42 ± 6.32 ng/ml in the NSCLC group and 14.12 ± 3.29 ng/ml in the control group (p <0.01) (Figure 1). The 95th percentile of DKK-1 values of the control group was 22.1 ng/ml, which was used as the cutoff value.

### Relation of serum DKK-1 to clinicopathological characteristics and survival analyses

**Figure 2:**
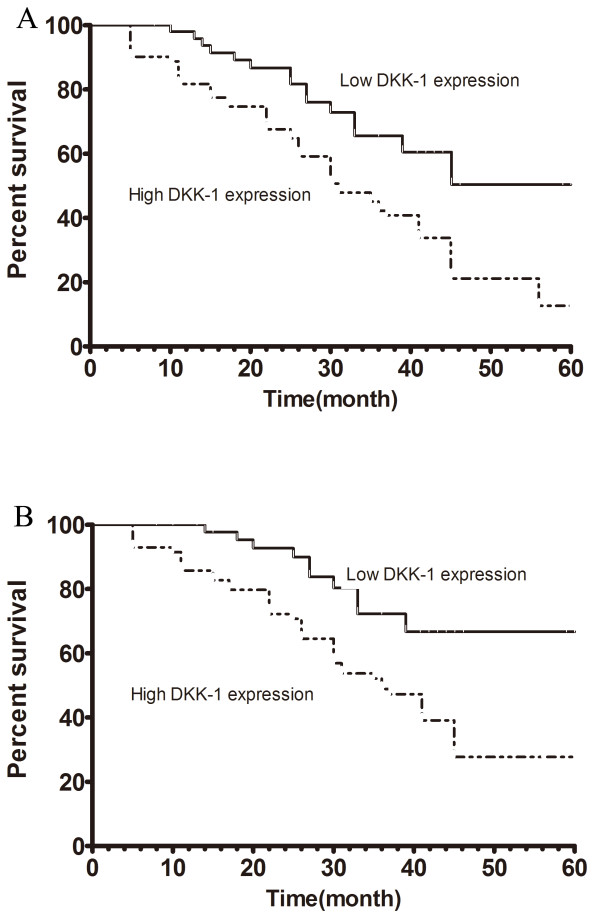
**Kaplan –Meier curves for patients with NSCLC. (A)**. Kaplan–Meier survival curve in relation to serum DKK-1 level in 150 patients with NSCLC. **(B)**. Kaplan–Meier survival curve in relation to serum DKK-1 level in 65 patients treated with surgery.

As shown in Table 2, serum DKK-1 level expression levels were significantly positively correlated with TNM stage (p = 0.009), lymph node involvement (p = 0.001), and distant metastases (p < 0.001). However, no significant correlation was observed between serum DKK-1 level and other clinicopathologic parameters, including age (P = 0.77), gender(P = 0.91), and smoking history (P = 0.81). We then detected whether the serum expression level of DKK-1 would be of any prognostic relevance in NSCLC. For this purpose, we performed survival analysis. As shown in Figure 2A, among all of the 150 patients with NSCLC, the patients with higher levels of serum DKK-1 had significantly poorer survival than those with lower expression levels of DKK-1, with a 5-year overall survival of 11.27% and 50.42%, respectively (P = 0.0027). Among the 65 patients treated with surgery, the patients with higher levels of serum DKK-1 had significantly poorer survival than those with lower expression levels of DKK-1, with a 5-year overall survival of 27.69% and 66.69%, respectively (P = 0.0033, shown in Figure 2B).

**Table 2:** Relation of serum DKK-1 to clinicopathological characteristics of 150 patients with non–small cell lung cancer

**Group**	**N (%)**	**DKK-1,ng/ml (mean[SD])**	**P value**
Gender			
Male	81(54%)	32.1(6.1)	
Female	69(46%)	30.2(5.9)	0.91
Age			
< 50 yr	15(10%)	29.9(3.6)	
50-70 yr	85(57%)	32.3(6.1)	
> 70 yr	50(33%)	31.1(4.2)	0.77
Smoking history			
Current	112(75%)	33.4(6.2)	
Former	29(19%)	31.1(5.7)	
Never	9(6%)	28.1(3.4)	0.81
TNM stage			
I	21(14%)	9.4(4.9)	
II	24(16%)	15.8(5.1)	
III	47(31%)	27.9(6.2)	
IV	58(39%)	49.1(6.7)	0.009
Lymph node			
No	103(69%)	24.3(6.6)	
Yes	47(31%)	57.9(3.9)	0.001
Distant metastases			
No	135(90%)	23.2(9.3)	
Yes	15(10%)	68.1(7.9)	<0.001

N: number; SD: Standard Deviation.

A Cox proportional hazards analysis was used to further evaluate the potential of serum DKK-1 expression level as a prognostic biomarker. Univariate survival analyses indicated that serum DKK-1 expression, TNM stage, lymph node involvement, and distant metastases were associated with prognosis, while gender, age, and smoking status were not associated with prognosis. In the multivariate Cox proportional hazards analysis, which included gender, age, smoking status, serum DKK-1 expression level, TNM stage, lymph node involvement, and distant metastases, high DKK-1 expression was independently associated with poor survival (P < 0.001; HR = 3.98; 95% CI =2.19-4.83, shown in Table 3).

**Table 3:** Multivariate analyses for prognostic factors in patients with NSCLC

**Variables**	**HR**	**95% CI**	**P value**
Age	1.31	0.79-1.46	0.81
Sex	0.91	0.88-1.36	0.79
Smoking history	1.98	0.97-2.13	0.06
TNM stage	2.39	2.13-3.89	0.001
Lymph node involvement	2.13	1.99-4.12	0.002
Distant metastases	5.97	3.12-9.33	<0.001
DKK-1 in serum	3.98	2.19-4.83	<0.001

CI, confidence interval; HR, hazard ratio.

## Discussion

As we know, the Wnt pathway plays an important role in development and in regulating adult stem cell systems, and aberrant activation of the Wnt signaling pathway is a major trait of many human cancers, including colorectal cancer, melanoma, NSCLC, leukemia, and bladder cancer [17]. A variety of cellular processes are mediated by Wnt signaling, including proliferation, differentiation, survival, apoptosis, and cell motility [18]. Wnts include negative and positive regulators, which act either intracellularly to modulate components of the signal transduction machinery or extracellularly to modulate ligand receptor interactions. Extracellular Wnt antagonists are composed of 5 families: the secreted frizzled-related protein, Wnt inhibitory factor 1, Xenopus Cerberus, Wise, and the DKK family [19].

The DKK family encodes secreted proteins, consisting of DKK-1, DKK-2, DKK-3, DKK-4, and a unique DKK-3-related gene, called Soggy. DKK-1, DKK-2, DKK-3, and DKK-4 contain two discrete cysteine-rich domains, in which the positions of 10 cysteine residues are supremely conserved among family members. DKK-1 binds to low-density lipoprotein receptor related protein-5/6 and blocks interaction with Wnt-1, resulting in β-catenin degradation and effects on proliferation [20,21]. Another study showed that DKK-1 functioned not only as an antagonist of the Wnt/β-catenin pathway but also as an agent that could upregulate other Wnt signaling pathways if the requisite Wnt/receptor combinations were available [22]. DKK-1 also can suppress cell growth and induces apoptotic cell death by activating the c-Jun N-terminal kinase pathway [23]. Previous studies have shown that the expression of DKK-1 was down-regulated significantly in human colon cancer, gastric cancer and melanoma, suggesting that DKK-1 may act as a tumor suppressor in these cancers and its role as an antagonist of Wnt signaling is lost in these cancers. However, paradoxically, DKK-1 has been found to be over expressed in hepatoblastoma and hepatocellular carcinoma, suggesting that DKK-1 may be feedback regulated by activated Wnt signaling pathway. It also means that the function of DKK-1 may differ depending on the cancer type.

Since DKK-1 is a secreted protein, serum level of DKK-1 and its prognostic potential have been investigated in several cancers. For example, Yang H et al. found that the mean serum level of DKK-1 in patients with early hepatocellular carcinoma was significantly higher than that in patients with cirrhosis, noncirrhotic chronic hepatitis B, benign liver tumors and healthy individuals (p < 0.001). The patients with a high serum DKK-1 level had a poorer overall survival (p = 0.028) and relapse-free survival (p = 0.045) than those with a low expression level [24]. Recently, Jiang T et al. found that the levels of serum DKK-1 were significantly increased in patients with cervical cancer compared with healthy women and patients with cervical intraepithelial neoplasia (p < 0.001). Further more, the expression level of DKK-1 in serum was correlated with lymphatic metastasis and tumor diameter in cervical cancer and associated with the prognosis of patients with cervical cancer [25].

In the present study, we investigated whether DKK-1 was secreted into the serum of patients with NSCLC. Our results showed that the levels of serum DKK-1 were significantly increased in patients with NSCLC compared with healthy controls. Further more, we found that serum DKK-1 level expression levels were significantly positively correlated with TNM stage, lymph node involvement, and distant metastases, suggesting that DKK-1 might be involved in the carcinogenesis and metastasis of NSCLC. More importantly, we proved that patients with a high expression of DKK-1 tended to have shorter survival than patients with lower levels, indicating that high DKK-1 level was a marker of poor prognosis for patients with NSCLC. However, in the present study, we have not investigated the detailed mechanism of DKK-1 in the carcinogenesis and metastasis of NSCLC.

In conclusion, our results showed that DKK-1 was overexpressed in NSCLC, and DKK-1 in serum was a good predictor of poor prognosis in patients with NSCLC. More researches are needed in the future to clarify the detailed mechanism of DKK-1 in the carcinogenesis and metastasis of NSCLC.

## Competing interests

The authors declare that they have no competing interests.

## Authors’ contributions

LLD and LYQ designed the study and drafted the manuscript; LLD, LYQ, LYC, XHZ, and YHL carried out the expertiments and performed the data analysis. All authors read and approved the final manuscript.
